# Optimal diagnosis and management of common nail disorders

**DOI:** 10.1080/07853890.2022.2044511

**Published:** 2022-03-03

**Authors:** Debra K. Lee, Shari R. Lipner

**Affiliations:** a Paul L. Foster School of Medicine, Texas Tech University Health Sciences Center El Paso, El Paso, TX, USA; b Department of Dermatology, Weill Cornell Medicine New York, NY, USA

**Keywords:** Nail disease, dermatology, brittle nail syndrome, onychomycosis, paronychia, nail psoriasis, longitudinal melanonychia, Beau’s lines, onychomadesis, retronychia

## Abstract

Nail conditions are not only aesthetic concerns, and nail changes may be a clue to an underlying systemic diseases or infection. Without timely treatment, nail diseases can continue to worsen and significantly impair performance of daily activities and reduce quality of life. Examination of the nails is essential at every medical visit, and may uncover important findings. Brittle nail syndrome, onychomycosis, paronychia, nail psoriasis, longitudinal melanonychia, Beau’s lines, onychomadesis and retronychia are common nail disorders seen in clinical practice. These conditions stem from infectious, inflammatory, neoplastic and traumatic aetiologies. Though each nail condition presents with its own distinct characteristics, the clinical findings may overlap between different conditions, resulting in misdiagnosis and treatment delays. Patients can present with nail plate changes (e.g. hyperkeratosis, onycholysis, pitting), discolouration, pain and inflammation. The diagnostic work-up of nail disease should include a detailed history and clinical examination of all 20 nail units. Dermoscopy, diagnostic imaging and histopathologic and mycological analyses may be necessary for diagnosis. Nail findings concerning for malignancy should be promptly referred to a dermatologist for evaluation and biopsy. Nail disease management requires a targeted treatment approach. Treatments include topical and/or systemic medications, discontinuation of offending drugs or surgical intervention, depending on the condition. Patient education on proper nail care and techniques to minimize further damage to the affected nails is also important. This article serves to enhance familiarity of the most common nail disorders seen in clinical practice. It will highlight the key clinical manifestations, systematic approaches to diagnosis and treatment options for each nail condition to improve diagnosis and management of nail diseases, as well as patient outcomes.

**Key messages**
- Nail disease is not only a cosmetic issue, as nail changes can indicate the presence of a serious underlying systemic disease, infection or malignancy.
- Nail pain and changes associated with NP are physically and emotionally distressing and may contribute to functional impairment and diminished quality of life.
- LM is a hallmark sign of subungual melanoma and this finding warrants further investigation to rule out malignancy.

## Introduction

The nail unit serves many important functions. It acts as a protective and mechanical tool of the distal digit. It also has aesthetic value, enhances sense of touch and is critical for picking up small objects. Disruption of this functional unit can cause considerable disability. Brittle nail syndrome, onychomycosis, paronychia, nail psoriasis (NP), longitudinal melanonychia (LM), Beau’s lines, onychomadesis and retronychia are common nail disorders seen in clinical practice. Detailed history and clinical examination are essential for an accurate diagnosis. Dermoscopy, imaging and histopathological and mycological testing may also be necessary for diagnosis. Although commonly considered minor aesthetic concerns, nail changes can be manifestations of serious diseases that require further investigation.

## Materials and methods

This review is based on peer-reviewed journal articles and guidelines indexed in PubMed. References cited by these articles were also reviewed for relevant publications.

## Results

Nail diseases are far more than cosmetic concerns and can greatly impact ability to perform daily activities. Clinical presentations of these nail conditions vary greatly and the ability to form a differential diagnosis can streamline diagnosis and initiation of treatment. Evaluation of the nails is important for assessing for possible underlying diseases.

## Discussion

### Brittle nail syndrome

Brittle nail syndrome is characterized by increased fragility of the nail plate resulting in splitting, peeling and friability. This condition almost exclusively involves the fingernails and affects up to 20% of the population. Women are affected twice as often as men [1]. Since the nail plate is a keratinized structure, factors altering nail plate production or directly damaging the nail plate can result in brittle nails [2]. The causes of brittle nails include normal ageing, infections, inflammatory diseases and occupational trauma or exposure to chemical agents [3].

### Idiopathic brittle nails

Brittle nails are characterized by disorganisation of keratin filaments, protein and lipid structure under electron microscopy. The crosslinking of keratin filaments between cysteine residues *via* disulphide bonds allow for adhesion between corneocytes and contribute to nail hardness [3]. Low lipid content reduces the nail’s ability to retain water, making it susceptible to splitting. Occupational and environmental exposures, including frequent contact with water or chemical agents, recurrent trauma to the nail plate and manicures also disturb cell adhesion and even modify keratin composition [4,5]. All of these changes contribute to the progressive dehydration of the nail plate and further contribute to nail brittleness.

**Figure 1.**
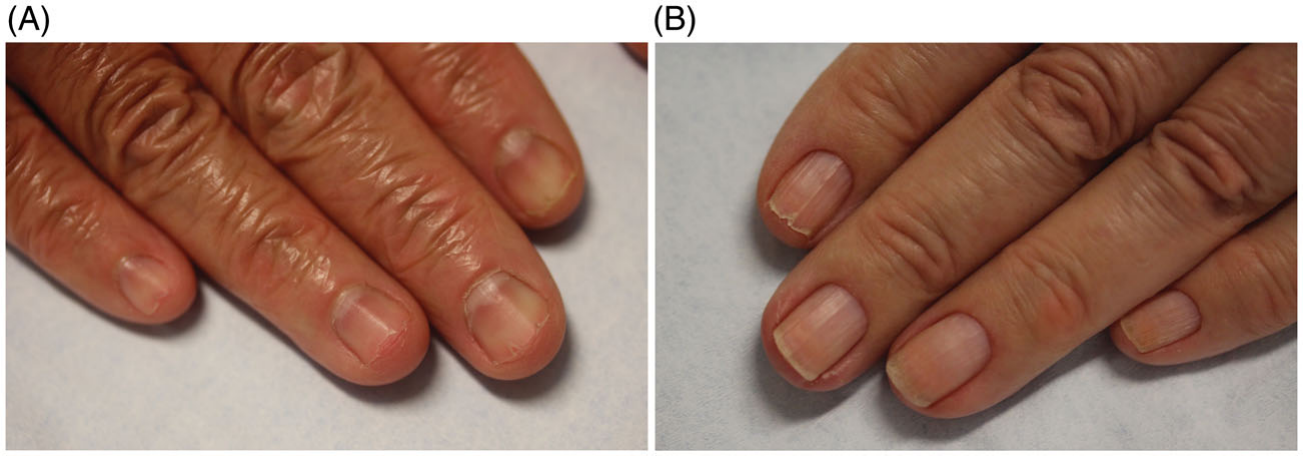
Clinical manifestations of brittle nail syndrome. (A) Lamellar onychoschizia. (B) Onychorrhexis.

Clinically, there are three types of nail fragility: lamellar onychoschizia, onychorrhexis and superficial granulation of keratin. Lamellar onychoschizia, also known as lamellar dystrophy, is characterized by the presence of fine horizontal layers that crack and peel easily from the free margin (Figure 1(A)). Impaired intercellular adhesion of the nail plate is thought to contribute to this condition [2,3,6]. This form of nail fragility is common in patients who wash their hands excessively (e.g. healthcare workers and homemakers) and those with lichen planus [3,5]. Onychorrhexis is defined as longitudinal splitting and fissuring of the superficial nail plate (Figure 1(B)). It is often seen among the elderly and in conjunction with onychoschizia. Its clinical presentation depends on the severity and degree of involvement of the nail matrix. It may result from an isolated split at the free edge of the nail plate that extends proximally [3]. Superficial granulation of keratin presents in the distal nail plate. It is characterized by white-yellow discolouration and striations. The keratins in the nail plate undergo an exfoliative process resulting in formation of patches. This condition is most commonly reported in patients who wear nail varnish often [3,5].

### Secondary brittle nails

Dermatological diseases, nutritional deficiencies and medications are associated with brittle nail syndrome (Table 1). Associated dermatological conditions include psoriasis, lichen planus, Darier’s disease and eczema. Typically, these conditions are diagnosed independently, but overlap in terms of nail fragility is frequently seen. NP is characterized by pitting of the nail plate, whereas lichen planus exhibits nail plate thinning [4,5]. Vitamin deficiencies, particularly in vitamins A–E, as well as other nutritional deficiencies in iron, zinc and selenium are contributors to brittle nails [2,7]. Drugs, including retinoids, antiretrovirals and chemotherapeutic agents are known to cause onychoschizia.

**Table 1.** Secondary causes of brittle nails.

Dermatological	Psoriasis
	Lichen planus
	Eczema
	Darier’s disease
	Alopecia areata
Nutritional deficiencies	Vitamins A, B, C, D and E
	Iron
	Zinc
	Selenium
Medications	Retinoids
	Antiretrovirals
	Chemotherapeutic agents
	Arsenic
	Penicillamine
Systemic	
Vascular	Peripheral artery disease
	Arteriosclerosis
	Raynaud’s disease
Hematological	Iron deficiency anaemia
	Polycythaemia vera
Endocrinological	Thyroid disease
	Hypothyroidism, hyperthyroidism
	Hypopituitarism
	Diabetes mellitus
	Osteoporosis
Other	Pregnancy
	Sarcoidosis
	Amyloidosis
	Gout
	Malnutrition
	Neuropathy
Infectious	Onychomycosis
	Pulmonary tuberculosis
	Bronchiectasis
	Syphilis
	Hepatitis B and C

Vascularisation and oxygenation of the nail matrix is essential for normal keratinisation. Therefore, vascular and haematologic diseases that directly affect oxygenation, such as peripheral artery disease, arteriosclerosis, Raynaud’s disease, iron deficiency anaemia and polycythaemia vera, are risk factors for brittle nails [1,4,5].

A number of endocrine and metabolic disorders are also associated with brittle nails. These include thyroid disease, hypopituitarism, diabetes and osteoporosis, which contribute to slow nail growth, leading to fragility [4]. Chronic infectious diseases like onychomycosis, pulmonary tuberculosis and syphilis also impair proper nail formation [3].

### Treatment

When nail brittleness is due to a systemic or dermatological disease, management of the underlying disease is first priority. Treating the underlying condition typically results in resolution of symptoms. When infectious aetiologies are suspected, the diagnosis is confirmed with clippings or cultures and the patient is treated with the appropriate antifungal or antibiotic. When nail brittleness is idiopathic, which encompasses the majority of these patients, a targeted approach is necessary. Detailed history and physical exam are often helpful in determining the cause of the patient’s symptoms. Patients should avoid exposure to water or chemical solvents by wearing cotton gloves under vinyl gloves for wet work. For dry work, use of heavy cotton gloves is recommended [3]. In patients with occupations involving repeated microtrauma to the nails, nails should be kept short to minimize damage and onychoschizia [4,8].

To date, there is limited evidence that oral supplementation with biotin (vitamin B7 or H) improves brittle nails. However, biotin is still widely recommended by physicians or self-prescribed by patients for brittle nails. Biotin has been associated with alterations in laboratory test results, including falsely low troponin levels with resulting myocardial infarction [9,10]. Emollients and humectants (glycerin and propylene glycol), especially those containing phospholipids, improve hydration to the nail [11,12]. Patients may also benefit from using nail hardeners, although caution is advised due to its potential to paradoxically worsen fragility and onychoschizia [1].

### Onychomycosis

Onychomycosis is a fungal nail infection caused by dermatophytes, moulds and yeasts. It is the most common nail disorder, with a worldwide prevalence of 5.5% and accounting for 50% of all nail disorders seen in clinical practice [13,14]. Onychomycosis is common in older individuals with 20% prevalence in the 60 years and older age group [15]. Prior nail trauma, history of tinea pedis, diabetes mellitus and immunosuppression are well-established risk factors for onychomycosis [14]. Hyperhidrosis, occlusive footwear and genetics can also predispose to onychomycosis risk.

### Clinical presentation

Patients with onychomycosis can experience pain while walking, difficulty fitting into shoes and social consequences [14,16]. In many cases, patients will defer treatment until infection becomes severe due to the misconception that the condition is only a cosmetic issue or will resolve without treatment [17]. Thorough examination of the nails even during routine office visits is crucial for this reason and can minimize treatment delay.

**Figure 2.**
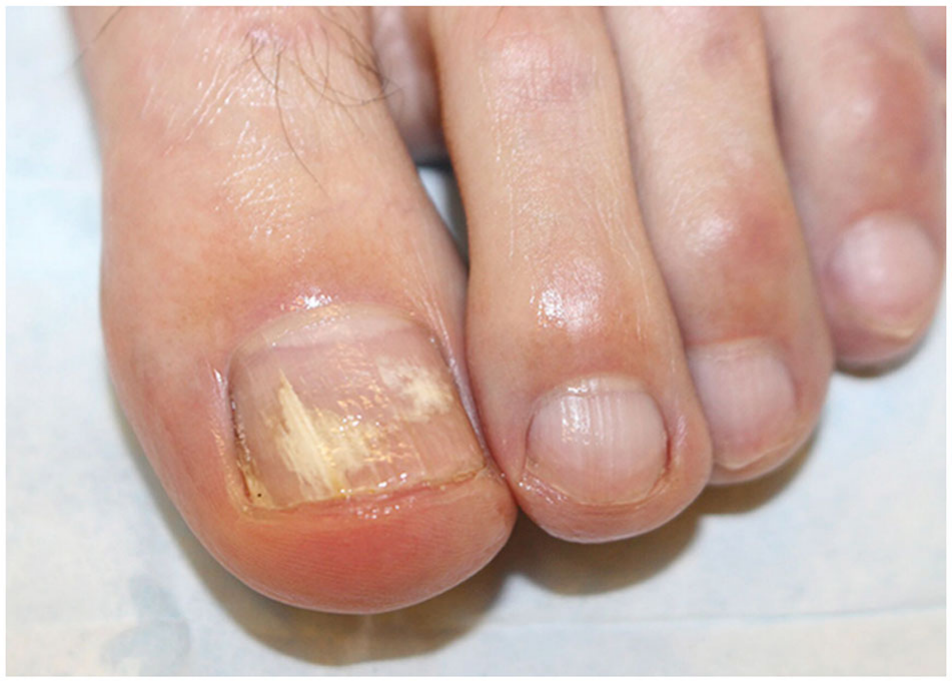
Dermatophytoma (DOI: 10.1016/j.jaad.2018.03.062, Permission for reuse of this image has been obtained from the copyright holder (Elsevier) and applies to publications with the Creative Commons license).

Onychomycosis commonly affects the toenails, with the great toenail most frequently involved. On physical exam, onychomycosis presents with subungual hyperkeratosis, characterized by build-up of keratinocytes under the nail plate, causing the nail plate to lift and detach distally (onycholysis) [17]. Over time, nail plate thickening and crumbling, and rarely, haemorrhages and shedding of the entire nail may occur [18]. White or yellow–brown discolouration and subungual debris may also be present [19]. Dermatophytomas are collections of concentrated fungal elements and present clinically as linear white or yellow streaks in the nail (Figure 2) [20].

**Figure 3.**
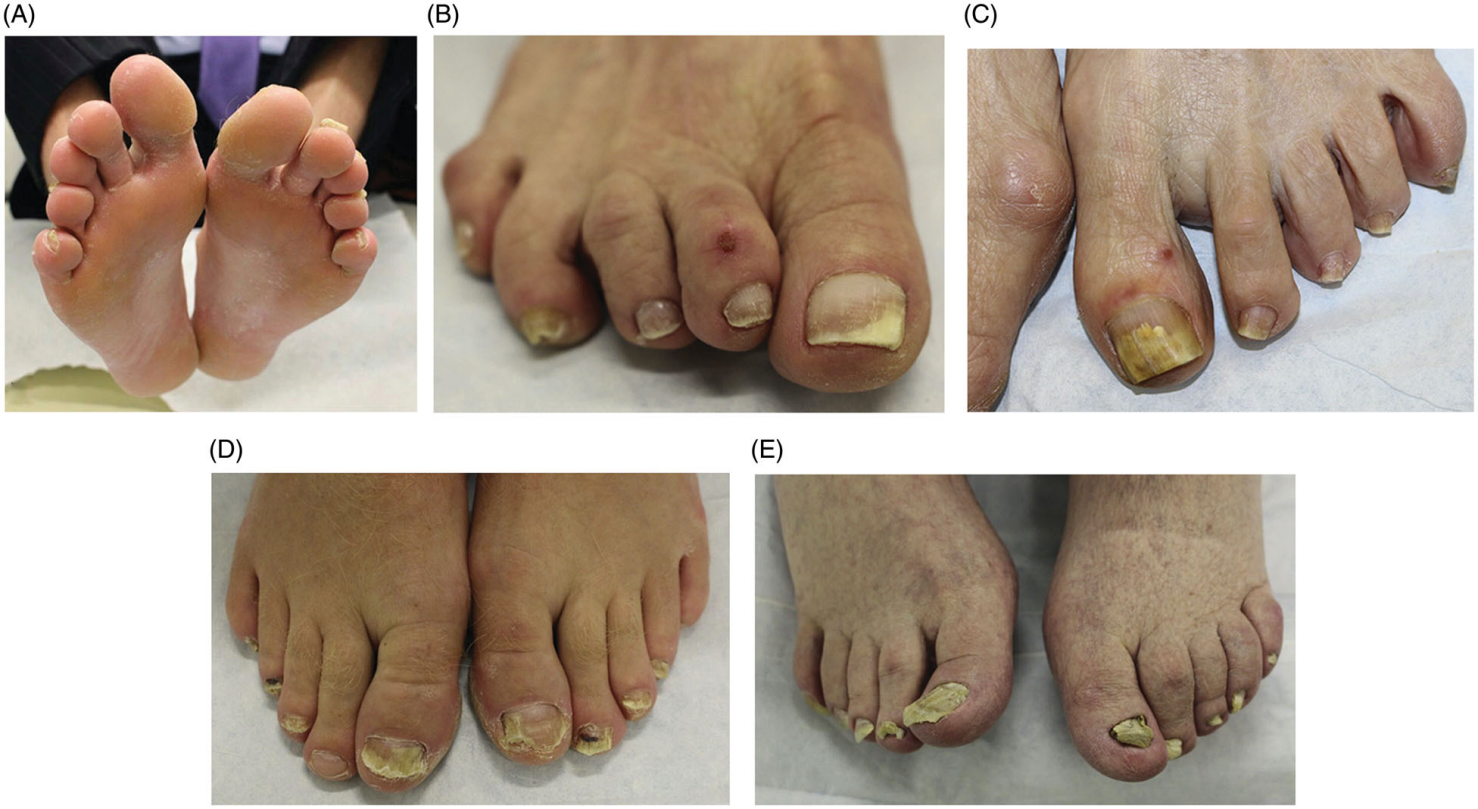
Clinical examination findings in onychomycosis. (A) Tinea pedis, scale on the plantar feet and web spaces. (B) Subungual hyperkeratosis and onycholysis of the right great toenail. (C) Yellow discolouration and onycholysis of the left great toenail. (D) Subungual hyperkeratosis and onycholysis in multiple toenails. (E) Severe onychodystrophy in multiple toenails. (DOI: 10.1016/j.jaad.2018.03.062, Permission for reuse of these images has been obtained from the copyright holder (Elsevier) and applies to publications with the Creative Commons license).

Onychomycosis is grouped into subtypes based on the pattern of fungal invasion. Distal lateral subungual is by far the most common subtype, characterized by spread of infection starting from the distal-lateral border of the hyponychium and proceeding proximally [21]. It is commonly associated with scale on the plantar feet and web spaces (tinea pedis; Figure 3(A)) and presents with nail plate discolouration, subungual hyperkeratosis and onycholysis (Figure 3(B–D)) [22,23]. Proximal subungual onychomycosis is a less common subtype. Infection begins under the cuticle and proceeds from the proximal nail plate to the distal nail plate. This subtype is associated with immunosuppression (e.g. HIV) when onset is abrupt and progresses rapidly [21,24]. White superficial onychomycosis appears as milky white, opaque patches that are easily scraped away from the superficial nail plate [25]. Endonyx onychomycosis involves the majority of the nail plate without nail bed involvement. Lamellar splitting and whitish discolouration without hyperkeratosis or onycholysis are hallmarks of this subtype [24]. Finally, total dystrophic onychomycosis is the most advanced form and is the result of chronic distal lateral and proximal subungual onychomycosis [21]. The nail bed is deformed and thickened, containing fragments of the nail plate (Figure 3(E)) [26].

### Diagnosis

Distinguishing onychomycosis from other nail diseases is paramount before initiating antifungal therapy. Failure to do so may lead to incorrect diagnoses, unwanted side effects and progression of disease [27,28]. Although detailed history and physical examination can help narrow the differential diagnosis, making the diagnosis without laboratory confirmation is highly inaccurate. Many nail conditions share clinical presentation similarities with onychomycosis (Table 2) [29,30].

**Table 2.** Differential diagnoses for onychomycosis.

Condition	Features
Psoriasis	Nail pitting, “oil staining,” splinter haemorrhages
Lichen planus	Longitudinal grooves or ridges, nail thinning
Lichen striatus	Longitudinal striae; usually affects one nail; common in children
Alopecia areata	Nail pitting, onychomadesis
Contact dermatitis	Erythematous patches involving nail folds, nail thickening, nail fragility
Paronychia	Inflammation of surrounding nail tissue, loss of cuticle; commonly caused by *Streptococcus, Staphylococcus,* or *Candida*
Verruca	Verrucous papules involving nail folds, longitudinal grooves
Trauma	Common with friction from footwear
Bowen’s disease, squamous cell carcinoma	Paronychia, onychodystrophy, nail plate discolouration, verrucous papule involving nail bed/nail fold, bleeding pain
Melanoma	Brown-black longitudinal band or red nodule, nail plate splitting, Hutchinson’s sign (hyperpigmentation involving the nail fold or hyponychium)

Dermoscopy is a quick, non-invasive and highly effective diagnostic tool to help differentiate onychomycosis from other nail disorders [31]. Jagged proximal borders with spikes as well as longitudinal streaks are common features seen on dermoscopy [32,33]. This should be considered the first step in the diagnostic work-up of onychomycosis, however mycological confirmation is still necessary.

Nail clipping or subungual debris samples are obtained with sterile clippers and curette, respectively. Microscopy using potassium hydroxide (KOH) 10%–20% solution can be performed in the office or sent to commercial laboratories. Successive KOH tests can be performed up to three times to improve sensitivity [34]. KOH is the most cost-effective option, but requires extensive expertise to interpret results. Alternatively, histopathological evaluation using periodic acid-Schiff staining allows for enhanced visualisation and greater sensitivity [35]. Infection is confirmed when hyphae are visualized. Identification of the fungal organism requires further testing. The presence of pseudohyphae is indicative of spores, representing yeast [14]. Fungal culture can identify the infecting organism and its viability [35]. Culture of the subungual debris is preferred over nail clippings because of decreased risk of bacterial or mould contamination [14]. A disadvantage of this technique is that fungal growth may take several weeks and false negatives are common [13]. Polymerase chain reaction is a newer method for identifying organisms. It is a DNA-based technique with increased sensitivity when compared to KOH and culture [36]. There may be false positives since all DNA may be amplified. Turnaround time is also rapid with results available within 48 h.

### Treatment

The goal of treatment is to eliminate the fungal infection and restore the nail to its normal state. Mycologic cure refers to a negative KOH microscopy and nail culture. Clinical cure is characterized as a clinically normal looking nail. Complete cure is defined as both a mycologic and clinical cure [18,37]. Patients should be advised that nail growth is slow and that continued improvement may occur even after treatment is completed [14,37]. In addition, disease recurrence is common without appropriate prophylaxis. After history and clinical examination, as well as, mycologic confirmation, treatment can be initiated.

Treatment options include oral antifungals, topical agents and devices. Systemic therapies (terbinafine and itraconazole) are often prescribed due to their accessibility, affordability and high efficacy [36]. Fluconazole may also be prescribed, but as off-label treatment for onychomycosis. While most patients do not experience side effects, headaches and gastrointestinal distress can occur in some patients [20]. However, elevated transaminases, hypertriglyceridaemia, neutropenia and drug–drug interactions are much less common, but serious adverse events associated with these agents. Before and during treatment, laboratory values should be monitored closely and medications carefully reviewed in patients that are at increased risk. Older adults, who may be more likely to have underlying conditions, including peripheral vascular disease and diabetes, as well as, polypharmacy can make treatment difficult. These conditions can impair wound healing and predispose patients to secondary infections [38]. Terbinafine is the treatment of choice in the elderly population, if there are no contraindications, as there are less associated side effects and drug interactions compared to itraconazole [39].

Topical antifungals are alternatives treatments for onychomycosis that have low risk of adverse systemic effects. They work by penetrating through or going under the nail plate to target the nail infection [40]. Limitations of topical antifungal treatment are long duration of treatment (minimum of 48 weeks) and generally lower efficacy [37]. Topical ciclopirox, efinaconazole and tavaborole are approved by the Food and Drug Administration for onychomycosis treatment [41,42]. Topicals may be used in milder adult cases of onychomycosis and in children, who have thinner nail plates and faster nail growth rates [43,44].

Nail debridement is an example of a physical treatment, which may be performed concomitantly with use of other antifungal agents and can improve treatment success rates [45,46]. Devices for treating onychomycosis include laser and photodynamic therapies. These therapies are expensive, have limited efficacy and have not been studied in large randomized clinical trials [20].

### Paronychia

Paronychia is defined as inflammation or infection of the proximal or lateral nail folds. This condition can be classified into acute, chronic or chemotherapy-associated paronychia (CAP) based on duration of symptoms and aetiology [47,48]. Infections are responsible for the acute subtype, while irritants and allergens are common causes of chronic paronychia [49]. Certain chemotherapeutic agents are responsible for CAP. Nevertheless, all subtypes involve a breach in the protective barrier in the nail fold. Women are more commonly affected than men. Common risk factors include trauma, use of artificial nails and manicuring, ingrown nails and nail biting [49].

## Aetiologies and clinical presentations

### Acute paronychia

Disruption of the protective nail barrier allows for invasion of pathogens and subsequent infections, which can occur following trauma. Nail biting, finger sucking, aggressive manicuring and hangnail manipulation are common causes of minor trauma to the fingernails [47]. In the toenails, acute paronychia is often due to ingrown nails [50]. Onset of symptoms typically occurs within a week from the initial trauma [51]. *Staphylococcus aureus* is the most common pathogen; however, most infections involve a mix of aerobic and anaerobic bacteria [52,53].

Acute paronychia presents with localized inflammation and nail fold pain, lasting no longer than 6 weeks. Only one digit is affected in most cases. Depending on the severity of infection, an abscess may also develop and spread to adjacent nail folds [50,54]. If left untreated, the abscess can further spread to the subungual region, with separation of the nail plate from the nail bed [55].

### Chronic paronychia

Inflammation of the surrounding nail folds lasting longer than 6 weeks is defined as chronic paronychia. It is not considered a primary infection. The cause of this condition is multifactorial, but is commonly due to repeated exposure to moisture and environmental irritants [50]. Homemakers, food handlers, dishwashers, swimmers and healthcare providers are at higher risk of exposure [49,55]. Patients with diabetes mellitus and immunosuppression are also likely to develop this condition [51]. *Candida albicans* is often cultured from patients with chronic paronychia, but it is thought to be a colonizer rather than a pathogen [26]. Less common causes of chronic paronychia include Raynaud’s disease, psoriasis, malignancies, retinoids and protease inhibitors [49,56,57].

Compared to acute paronychia, chronic paronychia presents with tenderness and swelling, but to a lesser degree. Patient may experience intermittent exacerbation of symptoms, after exposure to moisture or irritants [58]. Over time, ridging, discolouration and rounding of the nail plate may be seen [51]. The cuticle may separate from the nail plate or be totally absent [49,52]. More serious conditions like metastatic cancer, subungual melanoma and squamous cell carcinoma can present as chronic paronychia [54,59,60]. For this reason, the possibility of a neoplasm should be investigated if symptoms persist despite treatment.

### Chemotherapy-associated paronychia

CAP is a unique subtype of paronychia induced by chemotherapeutic agents. Epidermal growth factor receptor (EGFR) inhibitors (cetuximab, panitumumab, erlotinib and gefitinib) and taxanes (paclitaxel and docetaxel) are common offending medications. These drugs affect the differentiation of keratinocytes and inadvertently target the nail matrix [48]. CAP manifests about 4–8 weeks after chemotherapy initiation [61]. The great toenail is commonly affected with characteristic erythema, warmth and tenderness present. Patients may report difficulty-performing activities of daily living due to pain [62].

### Diagnosis

Paronychia is diagnosed based on history and clinical examination, which can help differentiate between acute and chronic conditions. The presence of an abscess can be confirmed using the digital pressure test. The physician applies gentle pressure to the distal volar aspect of the affected digit. If a larger than expected region of blanching occurs, an abscess is likely present [63].

In atypical presentations, ultrasonography, radiography and a complete blood count with differential are helpful for diagnosis [49]. An ultrasound of the infected digit can confirm the presence of an abscess. When foreign bodies, fractures and osteomyelitis are suspected, plain film radiographs are utilized. Laboratory tests are only indicated in cases of extensive cellulitis and lymphangitis [64]. Finally, a thorough review of the patient’s medication list is also advised when the diagnoses of chronic paronychia and CAP are being considered.

### Treatment

Conservative therapies are often sufficient for treatment of acute paronychia without abscess. Warm soaks in water, vinegar or antiseptic solutions (Burow solution, chlorohexidine, povidone-iodine) are effective and may promote spontaneous drainage [49,51,58,65]. The affected digit should be soaked for 10–15 min, multiple times a day [54]. Topical antibiotics can be added if minimal erythema is present. Mupirocin, gentamicin and bacitracin are safe and effective options, but there is higher incidence of contact dermatitis with bacitracin [66]. If infection persists, oral antibiotics with gram-positive coverage should be initiated. Additional anaerobic coverage should also be included when oral flora is suspected [50].

The presence of an abscess necessitates incision and drainage with culture and sensitivities. In cases with subungual abscess, complete nail avulsion is required [47]. Warm soaks after drainage can facilitate further drainage and prevent secondary infection. Oral antibiotics are unnecessary following the procedure, except for cases with cellulitis or positive bacterial cultures [67].

Chronic paronychia is managed with irritant and moisture avoidance; however, topical and systemic agents are also used [68]. Previously, antifungal agents were considered first-line for treating chronic paronychia. More recent studies have shown better success with topical steroids [69]. Topical antifungals can be used together with corticosteroids; however, this combination has not been shown to be superior to the use of topical steroid alone [51]. Systemic steroids can be considered for short durations in patients with severe presentations and failed previous treatments. In patients with refractory chronic paronychia, a trial of systemic antifungal treatment is recommended prior to surgical management [51].

Avoidance of trauma, irritants, moisture and restrictive shoes are recommended for CAP management [70]. Application of emollients and use of protective gloves during aggravating activities are also helpful [48]. Although there is no standardized treatment approach, there is some evidence that oral tetracyclines and topical steroids are beneficial in treating and preventing CAP [71,72]. In addition, daily moisturizer and sunscreen use may reduce the incidence of paronychia in patients receiving EGFR treatment [72]. In severe cases, dose adjustments may be necessary, but one study showed that drug concentration is unrelated to development of paronychia [61,73].

When non-surgical treatments fail, surgical intervention may be required for management of chronic paronychia. Eponychial marsupialization or complete nail avulsion is effective for treating chronic paronychia [74]. Excision of the affected nail fold is another surgical approach and is considered to be simpler and more effective than marsupialization [75]. Chronic paronychia involving a single nail that is resistant to treatment warrants exploration for possible underlying malignancy with a nail biopsy.

### Nail psoriasis

Psoriasis is a chronic inflammatory condition that can involve the skin, scalp, nails and joints [76]. Psoriasis most commonly affects the skin, and ∼50%–79% of patients have concurrent nail involvement. The estimated lifetime incidence of NP is 80%–90% [77–79]. NP is associated with a more severe cutaneous psoriasis disease course. Isolated NP has a significant disease burden and is a risk factor for psoriatic arthritis [80]. Patients often present with pain and functional impairment, contributing to diminished quality of life [81].

### Clinical presentations

**Figure 4.**
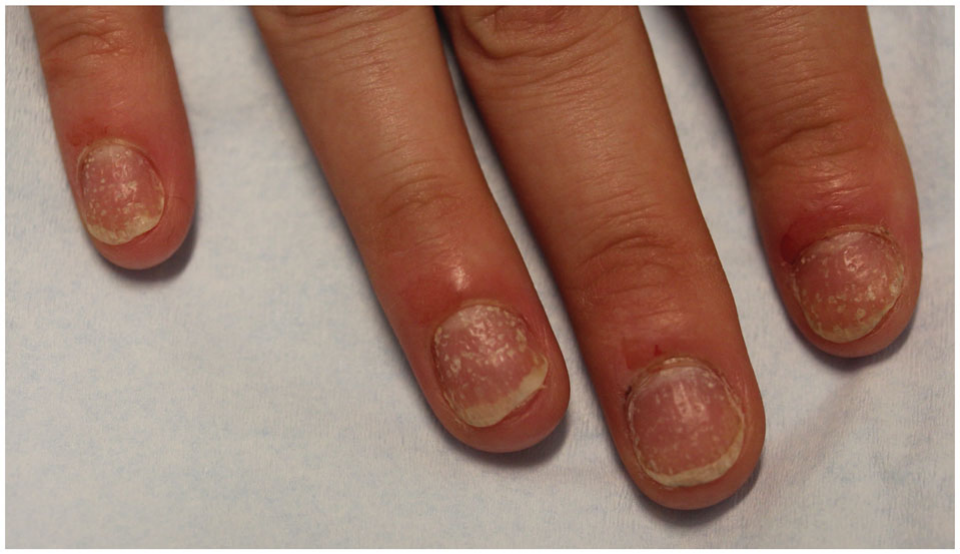
Nail pitting and onycholysis in right fingernails.

NP more frequently affects the fingernails compared to toenails. Clinical manifestations depend on the location of the nail unit that is affected—nail matrix or nail bed or both (Table 3). Nail matrix involvement results in nail pitting, thickening, crumbling, leukonychia and red spots in the lunula. Nail pitting is the most frequent and characteristic finding of NP. It is identified by small, sharply demarcated depressions on the nail surface, varying in size and depth (Figure 4) [82]. Although pitting can present in other nail conditions like eczema, alopecia areata and lichen planus, pitting in NP is typically deeper [76]. When the nail bed is involved, glycoproteins accumulate below the nail plate, producing a yellow discolouration known as the “oil drop” sign or salmon patch. If the oil spots extend distally or if psoriasis involves the distal nail bed, onycholysis may ensue [83,84]. Trachyonychia, splinter haemorrhages and subungual hyperkeratosis are also common manifestations of NP.

**Table 3.** Nail matrix and nail bed features of NP.

Nail Matrix	Nail Bed
Pitting	Oil drops/salmon patches
Leukonychia	Subungual hyperkeratosis
Nail plate thickening and crumbling	Onycholysis
Beau’s lines	Splinter haemorrhages
Red spots in lunula	
Trachyonychia	

Concomitant psoriatic arthritis is very common in patients with NP, and may be asymptomatic early in its disease course [85,86]. Patients may report significant disability performing daily tasks and impact on quality of life from disease burden and thus early detection is critical [87].

### Diagnosis

Diagnosis of NP is made based on history and clinical examination. Most patients will have signs of cutaneous psoriasis (well-demarcated erythematous plaques with silvery scale, often involving extensor surfaces). There is an average delay of nine years between initial presentation of cutaneous lesions and NP findings [88]. However, ∼5% of NP cases occur independently of cutaneous psoriasis [82].

Nail clippings with histopathological examination can help differentiate NP from other conditions, notably onychomycosis. Histopathology characteristically shows subungual parakeratosis with infiltration of neutrophils. Hyphae will be absent. A nail biopsy is rarely necessary for diagnosis except in difficult cases. A nail bed biopsy would be appropriate in the presence of the oil drop sign or onycholysis, whereas nail pitting requires a proximal matrix biopsy [83]. Dermoscopy, videodermoscopy and capillaroscopy are other tests used in the diagnostic evaluation of NP to better visualize nail changes.

The severity of NP can be determined using the nail psoriasis severity index, or NAPSI, which is based on dividing the nail unit into four quadrants and scoring for nail matrix and nail bed signs [83,89]. However, using this scoring system is time-consuming and does not always correlate well with clinical severity of NP [90]. Newer NP assessments have been proposed such as the Nijmegen-Nail psoriasis Activity Index tool, or N-NAIL, and the Nail Assessment in Psoriasis and Psoriatic Arthritis, or NAPPA, which incorporates quality of life measures and treatment expectations using two questionnaires. However, these assessments remain subjective and demonstrate limited clinical practicality [91].

### Treatment

Treatment of NP is based on number of nails affected, severity of disease, involvement of nail matrix or nail bed disease or both, skin involvement, joint involvement and impact on quality of life [92]. Successful treatment is also highly dependent on patient motivation and compliance given that NP is a chronic disease and that nail growth is slow [84]. Treatment options include topicals, intralesionals, systemics and biologics.

All NP patients should be counselled on general measures, including proper nail care and avoidance of activities that may further aggravate the disease. Prevention of mechanical trauma is highly emphasized, not only because it can worsen NP, but because it may also reduce the treatment efficacy [92]. These discussions increase patient satisfaction and compliance with treatment [93].

In cases of no or limited skin psoriasis with few-nail disease (NP affecting ≤3 nails) and nail matrix involvement only, intralesional steroid injections are considered first-line treatment. While painful, most patients do find the injections tolerable with proper technique. Subungual haematoma, short-term paresthaesia and atrophy are potential adverse side effects [94,95]. Alternatively, combinations of topical steroids (clobetasol propionate, betamethasone) and vitamin D analogues (calcipotriol) can be used; however, compliance may be poor [92].

In cases of no or limited skin psoriasis with few-nail disease and nail bed involvement, first-line therapy is topical steroids alone or in combination with topical vitamin D analogues. Other options include intralesional steroid injections, topical vitamin D analogues, topical retinoids or topical tacrolimus [92]. Patients are also advised to regularly clip the onycholytic nail plate for better efficacy [77]. If both nail matrix and bed are involved, intralesional injections and/or vitamin D analogues with topical steroids are the treatments of choice.

Systemic treatment is generally reserved for patients with ≥3 nails affected, extensive skin psoriasis, presence of psoriatic arthritis or when quality of life is severely impacted. Acitretin, cyclosporine and methotrexate are all options. Patients require routine monitoring while taking these medications due to risk of systemic adverse effects. Complete blood counts with liver, renal and lipid markers should be checked [96]. Acitretin can be used for more than six months, or until moderate improvement is noted. Cyclosporine is only recommended for short-term use. Methotrexate can be used for maintenance treatment at lower doses once there is moderate improvement at the full dose [92].

Biologic agents, like anti-TNF-α inhibitors infliximab, etanercept, adalimumab and golimumab; IL-12/23 inhibitor ustekinumab; IL-17 inhibitors secukinumab and ixekizumab; IL-23 inhibitor guselkumab; and the JAK 1/3 inhibitor tofacitinib, are examples of newer classes of medications used to treat psoriasis. Compared to traditional systemic drugs, biologics show more rapid and noticeable improvement with fewer side effects [82]. Some drawbacks to these options include their high cost and increased risk for infections.

### Longitudinal melanonychia

LM or melanonychia striata is characterized by a longitudinally oriented brown to black band extending the length of the nail plate. There are multiple causes of LM, including non-melanocytic and melanocytic aetiologies. Understanding these causes and their clinical presentations is crucial for preventing misdiagnoses and improving clinical outcomes.

## Aetiologies and clinical presentations

### Non-melanocytic causes

**Figure 5.**
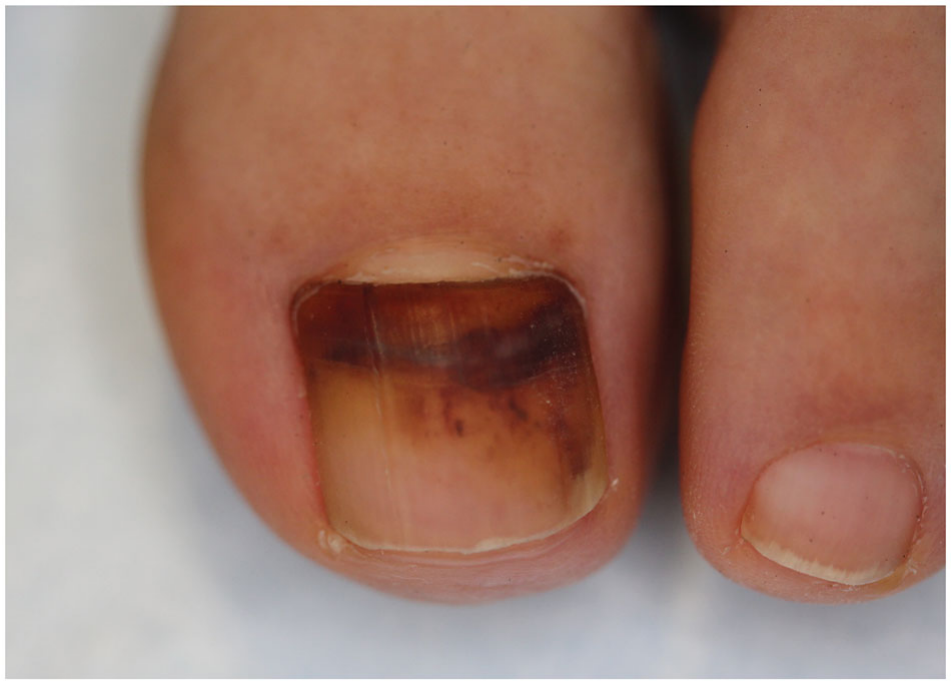
Subungual haematoma with onychomadesis.

Subungual haematoma is the most common cause of dark nail pigmentation. While some patients do recall trauma, many may not recall a prior incident. A true linear band is rare; however, a chronic subungual haematoma can mimic a longitudinal streak [97]. On dermoscopy, a homogenous pattern and globules are typically visualized. A distinguishing feature of a subungual haematoma is that the blood will grow out with nail plate growth (Figure 5). If there is no outgrowth of pigment, further evaluation for subungual melanoma is necessary. It should be noted, that the presence of blood does not necessarily rule out a nail unit melanoma, since melanomas may also bleed [98,99].

Other causes of non-melanocytic nail pigmentation include fungal melanonychia, green nail syndrome and exogenous pigmentation. *Trichophyton rubrum* and *Scytalidium dimidiatum* may cause fungal melanonychia, with *T. rubrum* producing a black pigment and *S. dimidiatum* causing diffuse brown pigmentation [100]. *Pseudomonas aeruginosa* is the causative bacteria in green nail syndrome, by production of pyoverdin and pyocyanin [101]. Exogenous pigmentation is caused by exposure to chemical agents, application of silver nitrate, tobacco, dirt and cosmetic products (henna, hair dyes). Exogenous pigment can be removed with alcohol or by gentle scraping with an 11 blade [99].

### Melanocytic causes

Pathogenesis of LM may be due to melanocytic activation or hyperplasia [102]. The former involves stimulating production of melanin or pigment and the latter is characterized by an increase in the number of melanocytes [102,103]. Clinical examination and dermoscopy can help to distinguish between these two subtypes, but nail biopsy is needed for definitive diagnosis.

### Melanocytic activation

**Figure 6.**
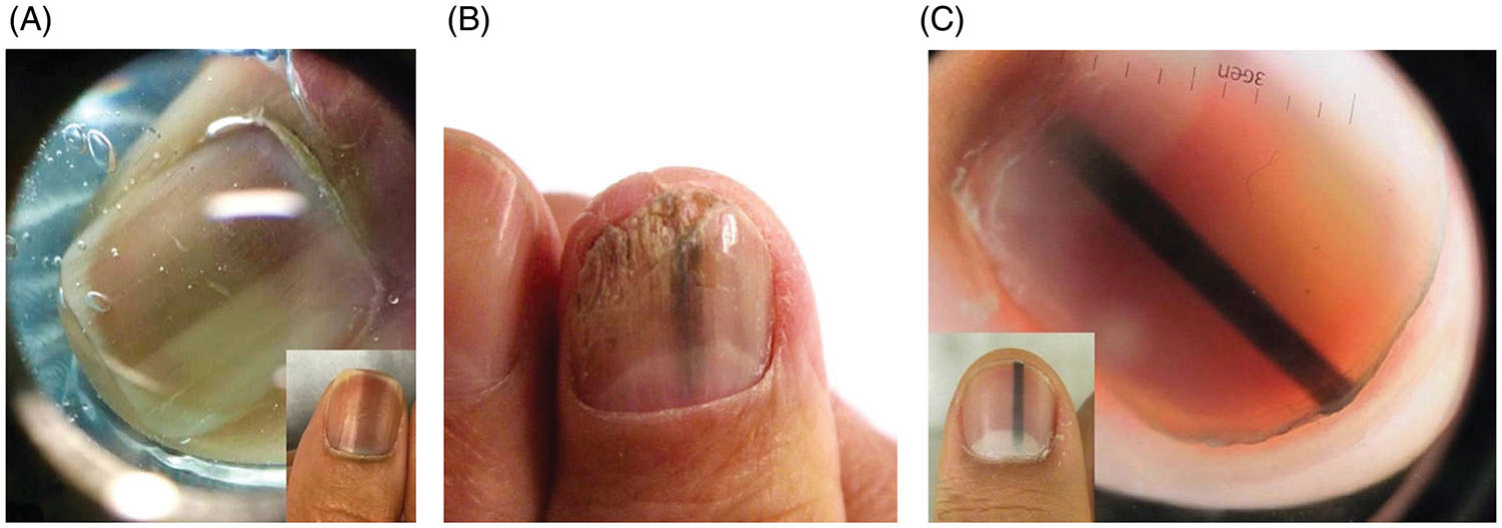
Clinical and dermoscopic findings of benign LM. (A) Ethnic melanonychia. On dermoscopy, grey background with multiple thin grey lines. (B) Melanocytic activation secondary to onychomycosis of the right thumbnail. (C) Junctional naevus. (DOI: 10.1016/j.jaad.2018.08.033, Permission for reuse of these images has been obtained from the copyright holder (Elsevier) and applies to publications with the Creative Commons license).

Melanocytic activation can be induced by physiological, dermatological, systemic and iatrogenic processes. Physiological causes include ethnic melanonychia and pregnancy. Darker pigmented individuals, namely Africans, Hispanics, Asians and Middle Easterners, are more likely to develop LM [102,104]. On clinical examination, there are often grey brown lines involving multiple nails. These bands most often appear in digits used for grasping (thumb, index finger, middle finger) or exposed to repeated trauma (great toe) [104]. On dermoscopy, a grey background with multiple thin grey lines is typical (Figure 6(A)) [105].

Dermatological causes of LM include onychomycosis, chronic paronychia, psoriasis, lichen planus and chronic radiodermatitis (Figure 6(B)) [102,106,107]. It is thought that chronic inflammation induces melanocytic activation. Systemic causes of LM include endocrine disorders, porphyria and genetic syndromes. Addison’s disease, Cushing syndrome and hyperthyroidism are among the most common endocrine disorders associated with melanonychia. Affected nails usually manifest with diffuse melanonychia comprised of multiple bands [98].

Iatrogenic melanonychia has been reported with phototherapy, chronic irradiation and drugs. Several digits are typically affected and sometimes the pigment resolves after cessation of the offending agent [108]. This subtype can present with transverse, diffuse or longitudinal hyperpigmented bands. However, transverse melanonychia is almost exclusively seen in drug-induced melanonychia [98]. Chemotherapeutics, like doxorubicin, bleomycin and cyclophosphamide and antimalarials, such as chloroquine, mepacrine and amodiaquine, are well-known agents associated with melanonychia.

### Melanocytic hyperplasia

Melanocytic hyperplasia is due to either a benign (naevus) or malignant (nail unit melanoma) aetiology. Nail matrix naevi can be congenital or acquired and are more common in children. A nail matrix naevus presents clinically as a brown to black longitudinal band typically involving one nail. Dermoscopy is characterized by with a brown background and longitudinal brown lines that are regular in terms of colour, width and spacing (Figure 6(C)) [105]. On histopathology, naevi are characterized by nests of melanocytes [98,109].

**Figure 7.**
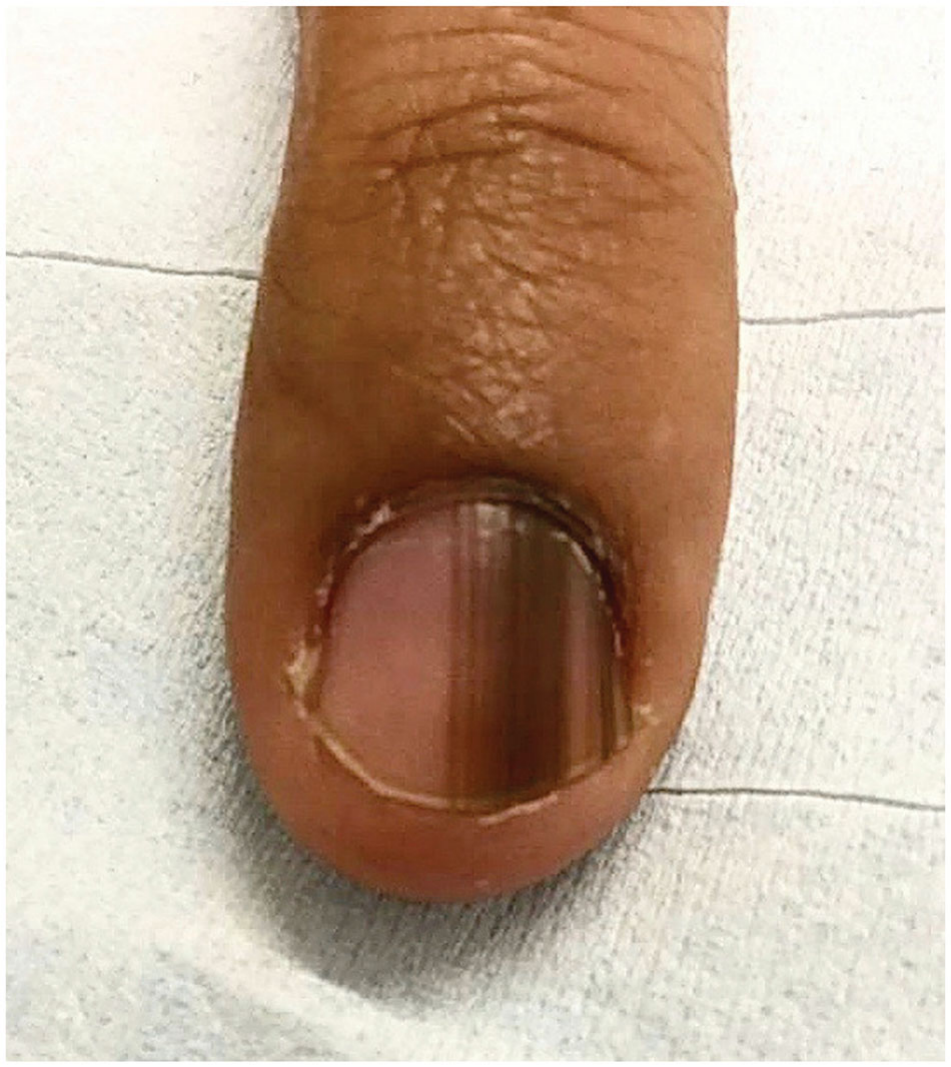
Subungual melanoma, 6 mm brown band. (DOI: 10.1016/j.jaad.2018.08.033, Permission for reuse of this image has been obtained from the copyright holder (Elsevier) and applies to publications with the Creative Commons license).

**Figure 8.**
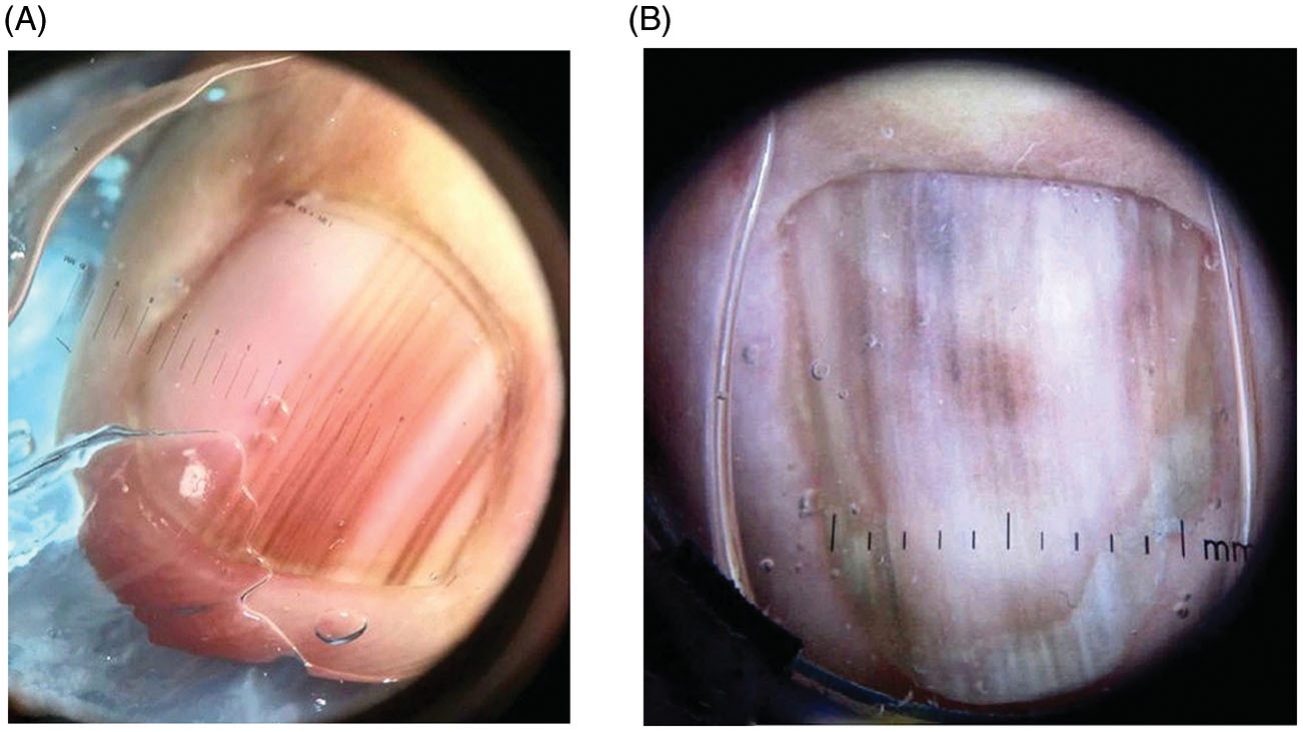
Dermoscopic findings of subungual melanoma. (A) Irregular colour, thickness and spacing without loss of parallelism. (B) Irregular colour, thickness and spacing with loss of parallelism. (DOI: 10.1016/j.jaad.2018.08.033, Permission for reuse of these images has been obtained from the copyright holder (Elsevier) and applies to publications with the Creative Commons license).

**Figure 9.**
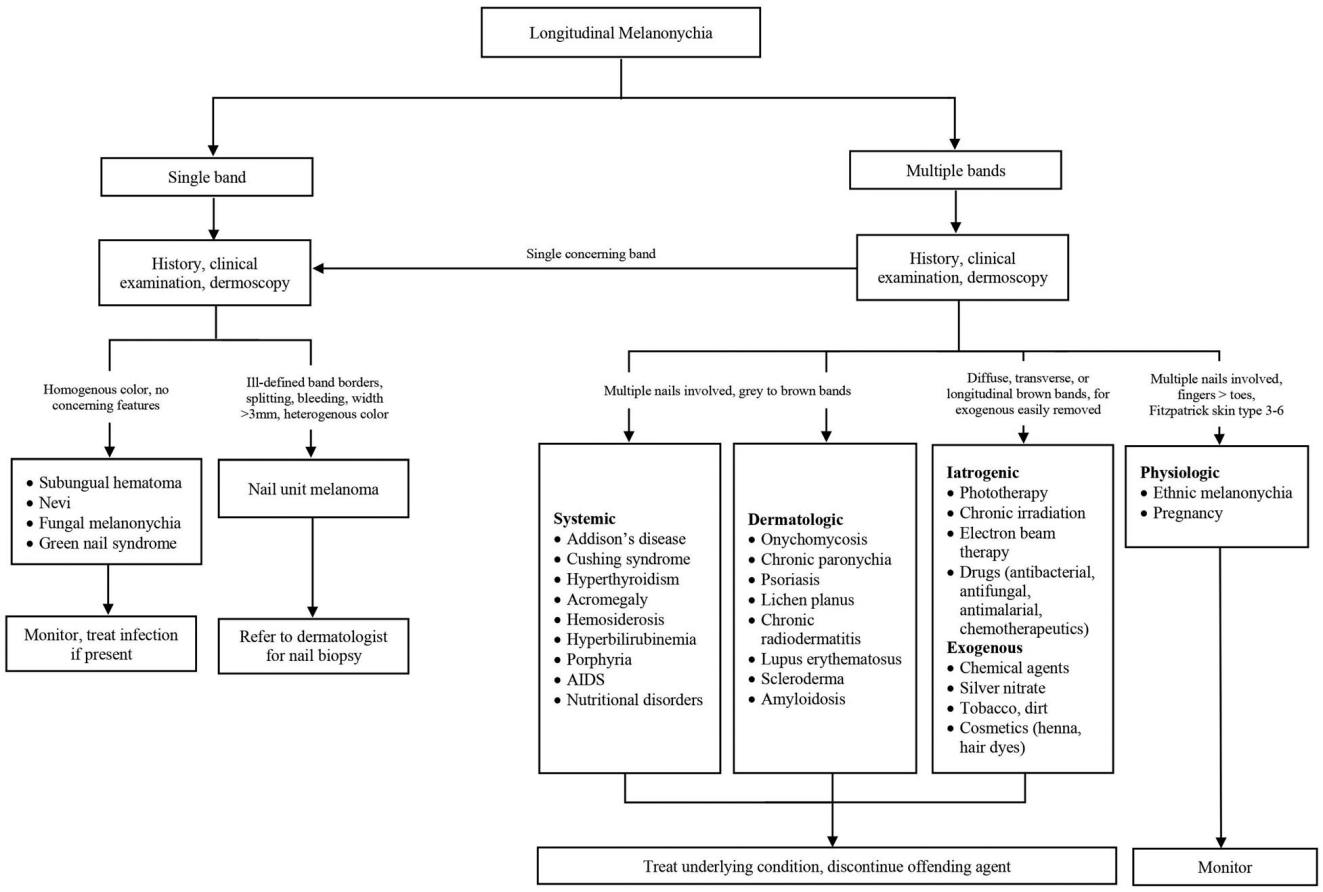
Systematic approach to LM.

LM is a hallmark sign of subungual melanoma and is frequently misdiagnosed, with an average of several years delay before diagnosis [97,110,111]. Therefore, subungual melanoma is associated with poor prognosis. It is most common in adults 50–60 years old. Clinical findings suggestive of malignancy include black band colour, variations in band colour, irregular band borders, periungual pigmentation (Hutchinson’s sign) and ulceration or haemorrhage (Figure 7) [20,98]. The pyramid sign, when the proximal band end is wider than the distal end, creating a triangular shape, is rarely seen, but is a poor prognostic sign [106]. Although the majority of melanonychia cases are benign, patients presenting with LM should always be investigated for subungual melanoma [103]. The ABCDEF rule has been suggested as an aid for diagnosis of subungual melanoma, and is markedly different than a similarly named cutaneous melanoma mnemonic. The A stands for *age* at presentation (between fifth and seventh decade); B stands for *brown or black* colour and *band breadth* of 3 mm and greater; C stands for *change* in size or morphology; D is for the *digit* involved; E is for *extension* of the pigment onto the nail fold; and F represents *familial* or personal history of melanoma or dysplastic naevi [112]. This method, although helpful, has not been validated and may not reliably distinguish malignant from benign LM [113]. On dermoscopy, a subungual melanoma typically has a brown background with irregularities in terms of colour, thickness and width (Figure 8(A,B)) [105]. In these instances, prompt referral to a dermatologist and nail matrix biopsy is required (Figure 9).

### Management

If history and clinical examination suggest a benign LM aetiology, treatment of LM is initiated based on underlying pathology. Treating the associated systemic disease or infection, discontinuing the offending drug and avoiding nail trauma are initial recommendations based on the cause. Benign causes of LM can be managed with routine follow-up. Detailed documentation and photographs are also helpful for monitoring. Patients should be educated on performing self-nail examinations and report any changes in colour or morphology of the band [98]. In patients with subungual melanoma that are *in situ*, en bloc excision is the preferred approach to preserve patient functionality and morbidity. For invasive melanomas, digit amputation is required, as well as, consultation with a medical oncologist.

### Beau’s lines, onychomadesis and retronychia

Beau’s lines, onychomadesis and retronychia are three nail disorders that are thought to lie on a continuum and share a common pathophysiology. For all three conditions, there is an insult to the nail matrix, which slows or halts nail plate production. Despite this shared pathophysiology, all three disorders most often present independently, but two to three can also occur concurrently [114]. Medication use, infection, systemic disease and trauma are common aetiologies [115]. Extent of trauma or severity of systemic insult to the nail matrix, duration of nail growth cessation and direction of growth are determinants of the clinical presentation. Differentiation of these three nail disorders from each other and from other nail conditions can expedite treatment and improve prognosis.

## Clinical presentation and etiologies

### Beau’s lines

**Figure 10.**
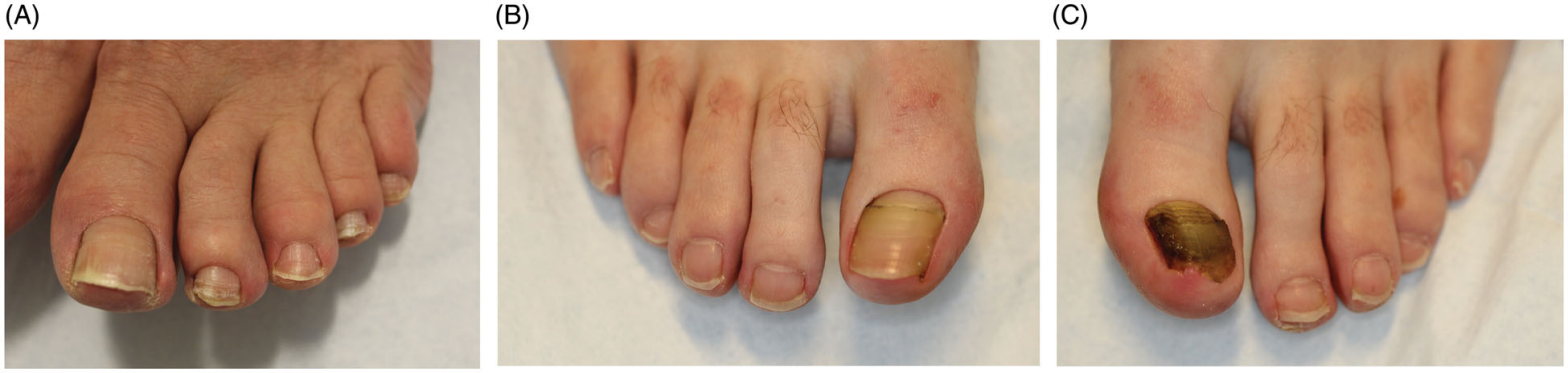
Clinical presentations of Beau’s lines, onychomadesis and retronychia. (A) Beau’s lines on the left toenails. (B) Onychomadesis of the left great toenail. (C) Retronychia of the right great toenail.

Beau’s lines are caused by transient decrease in mitotic activity of keratinocytes in the proximal nail matrix. This results in a thinner nail plate, creating a characteristic transverse groove (Figure 10(A)). The groove does not typically extend to the entire width of the nail plate. On average, fingernails and toenails grow at rates of 2–3 and 1 mm per month, respectively [20]. This can help estimate the timeframe of the insult. Further, the depth of the depressions correlates directly to the degree of damage of the nail matrix, while the width signifies the duration of insult [116]. While there are many causes of Beau’s lines, the most common is drug use, notably chemotherapeutics [117]. Drug-induced Beau’s lines are usually seen in all 20 nails and appear two or three weeks after initiating therapy [116]. Chemotherapeutic drugs inadvertently target tissues of high mitotic activity, such as the nail matrix. Shorter treatment durations with high doses and combination chemotherapy (e.g. docetaxel-cisplatin-fluorouracil) are more likely to cause Beau’s lines [116,118,119]. The nail pattern coincides with the timing between each chemotherapy cycle. The lines correspond to the beginning of each treatment cycle and the distance between the lines is proportional to the time interval between cycles [119]. Retinoids, radiation therapy, carbamazepine, cloxacillin, dapsone and itraconazole are medications that are also associated with Beau’s lines [120–123].

A number of infectious and systemic diseases have been associated with Beau’s lines. Diphtheria, syphilis, measles, mumps, malaria, typhoid fever, scarlet fever and hand-foot-mouth disease are among the many infectious aetiologies [114,123,124]. Acute systemic diseases like severe sepsis, rheumatic fever and myocardial infarction can cause Beau’s lines. Chronic conditions associated with Beau’s lines include uncontrolled diabetes mellitus, postpartum hyperparathyroidism, telogen effluvium and Raynaud’s disease.

Trauma-induced Beau’s lines are typically unilateral. A transverse depression involving a single nail suggests localized nail trauma, though injuries to the ipsilateral hand, wrist and elbow can also lead to Beau’s lines [125]. It is thought that temporary reduction of blood supply to the nail matrix following a trauma produces the nail changes [123]. Nerve injury from fractures and carpal tunnel syndrome also contribute to the development of Beau’s lines [126–128]. These injures are usually seen proximal to the nail fold, but distal injuries have also resulted in transverse indentations of the nails in some cases [129]. Limb immobilisation from casts or splints can further promote development of unilateral Beau’s lines, due to reduced nail growth associated with immobilisation [123,130,131].

### Onychomadesis

Onychomadesis is defined as a complete separation and eventual shedding of the nail plate (Figure 10(B)). It is a more extreme presentation of Beau’s lines and develops about four to eight weeks after the initial insult [132]. Complete loss of continuity with the matrix can occur when the inciting event is severe enough to cease nail production. If the subsequent depression that develops reaches a maximum depth, the nail plate will separate from the matrix [125,133]. As the proximal nail plate grows out, it undermines the distal plate, wedging it upward. Over time, the distal nail plate will shed [115]. In severe cases, inflammation and granulation tissue can be noted in the lateral nail folds [132].

Similar to Beau’s lines, onychomadesis is associated with medication use, infection and acute severe diseases. Cytotoxic chemotherapeutics, retinoids and antiepileptics are common medications likely to induce onychomadesis.

Infectious aetiologies include hand-foot-mouth disease, varicella infection and chronic paronychia [133]. These infections are commonly seen in young children. An increasing number of cases of hand-foot-mouth disease and onychomadesis has been observed worldwide [134–138]. Nail changes typically present at later stages of the disease and are associated with multiple Coxsackie viral serotypes (A6, A10, A16, B1 and B2) [124,138]. The exact mechanism is not known, but it is hypothesized that inflammation around the nail matrix causes shedding of the nail plate. Another possible cause is direct damage to the nail matrix from viral replication itself [136].

Onychomadesis has been reported in several major acute illnesses like Stevens-Johnson syndrome and Kawasaki disease [133,139,140]. It may also be seen in neonates who are subject to intrauterine or birth trauma (e.g. maternal infections, breech position) [141]. Autoimmune diseases, such as severe Guillain-Barré syndrome, pemphigus vulgaris and alopecia areata, are well-recognized conditions also associated with onychomadesis [142–144].

Although rare, idiopathic onychomadesis has been reported. This subtype is further classified into familial or sporadic [145]. Familial patterns of onychomadesis suggest an autosomal dominant inheritance pattern [146]. The sporadic variant can vary from seasonal shedding to random episodes of shedding. Though this form of onychomadesis is termed idiopathic, it is believed that microtrauma plays an important role [145].

### Retronychia

Retronychia is characterized by ingrowing of the nail plate into the proximal nail fold (Figure 10(C)). It is caused by disruption of the nail’s longitudinal growth. Retronychia commonly affects young adults, predominantly females, and is more frequent in toenails (great toe) than fingernails [147–149]. There are two stages of retronychia: the early and late stages [148]. The early stage shows interrupted nail growth, yellow discolouration and exudate formation underneath the nail fold. In the late stage, paronychia can present along with elevation of the proximal nail fold from edoema. The presence of granulation tissue between the proximal and lateral nail folds is another common finding.

Repeated microtraumas (e.g. jogging, hiking, ill fitted shoes) and a single major trauma are common reported triggers for retronychia [148]. Systemic conditions, like arthritis and thrombophlebitis, have also been associated with the development of retronychia [115]. Normally, a new nail plate will grow beneath the old plate to push it out. However, in retronychia, the new nail plate is still partially attached to the old plate and fails to move distally. The new plate can still pass under the old plate, pushing it upwards. As this process repeats, new nail plates will stack on each other, with the oldest nail plate on top. The sharp proximal edge of the old nail plate is continually pushed upwards into the ventral portion of the proximal nail fold [115,150]. As a result, patients may experience inflammation and pain. Walking can exacerbate the trauma between the proximal nail fold and plate as the footwear presses downward.

Retronychia is a relatively unrecognized nail condition, which can lead to misdiagnoses and delayed treatment. On average, symptoms can persist for months to years before it is correctly diagnosed [151]. Retronychia is commonly misdiagnosed as a paronychia. Patients may present with a history of unresolved chronic paronychia despite treatment with antibiotics and antifungals. Further evaluation often reveals paronychia secondary to underlying retronychia. Other differential diagnoses are onychomycosis, subungual tumours, nail psoriasis, verruca and arthropathies [151]. In recent years, there have been an increasing number of reports describing this nail condition.

### Diagnosis

Beau’s lines, onychomadesis and retronychia are clinical diagnoses. Distinct nail changes can be noted by inspecting the nail plate. The presence of transverse depressions or nail plate shedding can help differentiate between Beau’s line and onychomadesis. Retronychia will present with overlapping layers of nail plates. Patients should always be evaluated for recent history of severe disease, exposure to certain medications and trauma. A review of viral illnesses should be considered up to two months prior to initial nail changes [152].

For onychomadesis and retronychia, ultrasound imaging can help confirm the diagnosis by better visualising the defect under the proximal nail fold [132,151]. It may also help rule out conditions like subungual tumours or reveal abscesses. For onychomadesis, two nail plate fragments (proximal and distal) are usually seen. Ultrasound imaging can also determine an approximate timeframe for when the initial insult occurred. This is particularly useful for investigating a causal agent when multiple, continuous factors are being considered [132]. In retronychia, ultrasound imaging will reveal a thickened nail plate beneath the proximal nail fold. The presence of two or more superimposed nail plates is a confirmatory finding [148]. Further, colour Doppler ultrasounds can be used as an adjunct to identify areas of blood flow. This can help determine if a more invasive treatment option, including nail plate avulsion, is necessary.

### Treatment

Beau’s lines and onychomadesis are self-limiting nail conditions with excellent prognoses. There is no specific treatment, but patient education is important. Patients should be reassured that observation will most likely result in complete resolution of the conditions as long as the nail matrix is not permanently damaged [115]. The transverse depressions will progress distally with normal nail growth and disappear at the free nail edge when clipped off.

Patients should be educated on avoiding damage to the proximal nail plate by keeping nails short, in addition to treating underlying systemic diseases or discontinuing offending medications. Concerns over Beau’s lines are generally cosmetic. Patients may feel dissatisfaction with the physical appearance of their nails. Gel polish may be applied to fill in the nail plate grooves, which results in improved nail appearance and increases patient satisfaction [153]. For onychomadesis, there is reported treatment success with topical medications of 40% urea or halcinonide 0.1% in a few cases [154,155]. However, these treatments may not be universally effective. In advanced cases of onychomadesis that cause pain and impaired daily functioning, removal of the nail plate is necessary [156].

If retronychia is diagnosed in the early stage, conservative management can be helpful. Patients should be advised of wearing properly fitted shoes to reduce the occurrence of repeated microtraumas. Treatments like taping, orthosis and topical steroid application have also been beneficial in mild cases of retronychia. Strategically wrapping an adhesive around the affected digit may prevent retrograde movement against the proximal nail fold and allows for the nail to grow distally [150]. Though conservative treatments are efficacious, recurrence is seen in up to 16% of cases [148].

If conservative treatment fails, total or proximal nail plate avulsion is the treatment of choice for retronychia [157]. Avulsion of the nail can be either surgical or chemical. The surgical approach is fairly quick and provides rapid pain relief. Disease recurrence is rare with surgery when the patient has had the condition only a few months or less. Postsurgical complications include nail bed retraction and micronychia. This is due to loss of counter pressure from the nail plate, leading to expansion of the distal pulp and hyperkeratosis [148]. Chemical avulsion is an alternative to surgery, involving application of a topical ointment composed of 50% urea and salicylic acid 10% in white petroleum [158]. It has proven efficacious in some patients, but more investigation is required to confirm its reproducibility.

Nail changes are not mere cosmetic concerns. Disruption of the nails can lead to significant impairment in daily functioning, diminishing quality of life. Evaluation of the nails can provide insight into many systemic diseases and infections. Knowledge of the clinical manifestations in common nail disorders can streamline diagnosis and treatment and prevent further nail impairment. Treatment should involve patient education to avoid further damage to the nails as well as a targeted approach based on the aetiology.

## Data Availability

Data sharing is not applicable to this article as no new data were created or analysed in this study.

## Abbreviations

CAP: chemotherapy-associated paronychia
EGFR: epidermal growth factor receptor
IL: interleukin
JAK: Janus kinase
KOH: potassium hydroxide
LM: longitudinal melanonychia
NP: nail psoriasis
TNF: tumour necrosis factor
